# Superconducting Triplet Rim Currents in a Spin-Textured Ferromagnetic Disk

**DOI:** 10.1021/acs.nanolett.1c04051

**Published:** 2022-03-03

**Authors:** Remko Fermin, Dyon van Dinter, Michel Hubert, Bart Woltjes, Mikhail Silaev, Jan Aarts, Kaveh Lahabi

**Affiliations:** † Huygens-Kamerlingh Onnes Laboratory, Leiden University, P.O. Box 9504, 2300 RA Leiden, The Netherlands; ‡ Department of Physics and Nanoscience Center, University of Jyväskylä, P.O. Box 35 (YFL), FI-40014 Jyväskylä, Finland; § Computational Physics Laboratory, Physics Unit, Faculty of Engineering and Natural Sciences, Tampere University, P.O. Box 692, FI-33014 Tampere, Finland

**Keywords:** Superconductivity, Ferromagnetism, Magnetic texture, Triplet Cooper pairs, Usadel theory

## Abstract

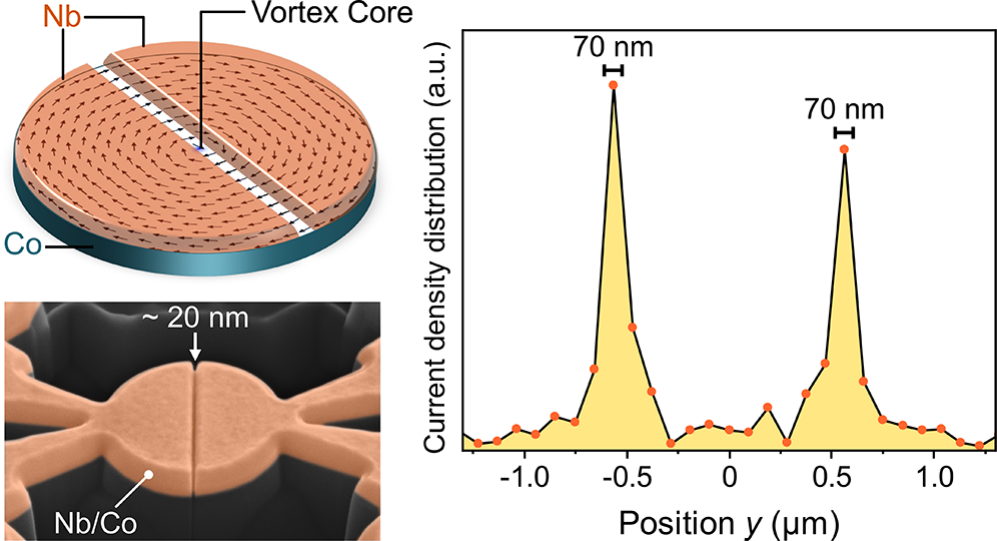

Since the discovery of the long-range superconducting proximity effect, the interaction between spin-triplet Cooper pairs and magnetic structures such as domain walls and vortices has been the subject of intense theoretical discussions, while the relevant experiments remain scarce. We have developed nanostructured Josephson junctions with highly controllable spin texture, based on a disk-shaped Nb/Co bilayer. Here, the vortex magnetization of Co and the Cooper pairs of Nb conspire to induce long-range triplet (LRT) superconductivity in the ferromagnet. Surprisingly, the LRT correlations emerge in highly localized (sub-80 nm) channels at the rim of the ferromagnet, despite its trivial band structure. We show that these robust rim currents arise from the magnetization texture acting as an effective spin–orbit coupling, which results in spin accumulation at the bilayer–vacuum boundary. Lastly, we demonstrate that by altering the spin texture of a single ferromagnet, both 0 and π channels can be realized in the same device.

## Introduction

The appearance of localized supercurrents at the edges of a Josephson junction is generally attributed to the topology of the electronic band structure and edge states.^1^ Edge states and the accompanying edge currents are typically found in ultraclean systems such as 2D electron gases,^2^ nanowires,^3^ and graphene.^4^ Here, we report the emergence of highly localized (sub-80 nm) spin-polarized supercurrents at the rim of disk-shaped Josephson junctions with a diffusive ferromagnetic barrier (Co). As we demonstrate, however, the rim currents are not related to the electronic band structure but rather a direct result of the interactions between spin-triplet Cooper pairs and the nontrivial spin texture of the ferromagnet.

At the interface between a superconductor and a ferromagnet, short-range triplet (SRT) Cooper pairs with zero spin projection emerge naturally via spin-mixing of singlet pairs and decay over a few nanometers (ξ_F_(Co) ∼ 3 nm ^5^) inside the ferromagnet. Long-range triplet (LRT) pairs can, on the other hand, propagate over substantially larger distances.^6,7^ Half-metallic systems can even show the LRT proximity effect over hundreds of nanometers.^8−11^ Due to their spin polarization, the LRT Cooper pairs can provide the means to combine the absence of Joule heating and decoherence with the functionality of spintronic devices.^12,13^ However, the controlled generation of LRT currents has proven to be a demanding process, commonly realized in complex superconductor–ferromagnet (S–F) hybrids, involving multiple F layers with noncollinear magnetization.^14−24^ Furthermore, a substantial body of research considered the possibility of generating and controlling LRT correlations using spin-textured systems, such as domain walls^6,25−28^ and vortices.^29,30^ However, the experimental evidence to verify such models remains scarce.^31^ In other recent developments, it was suggested that theoretically spin mixing can also be achieved by spin–orbit coupling (SOC).^32−36^ This led to researching long-range proximity effects with Josephson junctions containing heavy metal interlayers.^37−43^ In addition, recent studies suggest that spin–orbit coupling (SOC) can lead to spin accumulation at the edges of Josephson devices^35,44,45^ and, in some cases, generation of LRT currents.^46−49^ At present there is a complete lack of experiments that can examine the influence of SOC on LRT transport. As a consequence, the interplay between triplet pairing and magnetic texture as well as SOC remains elusive.

**Figure 1:**
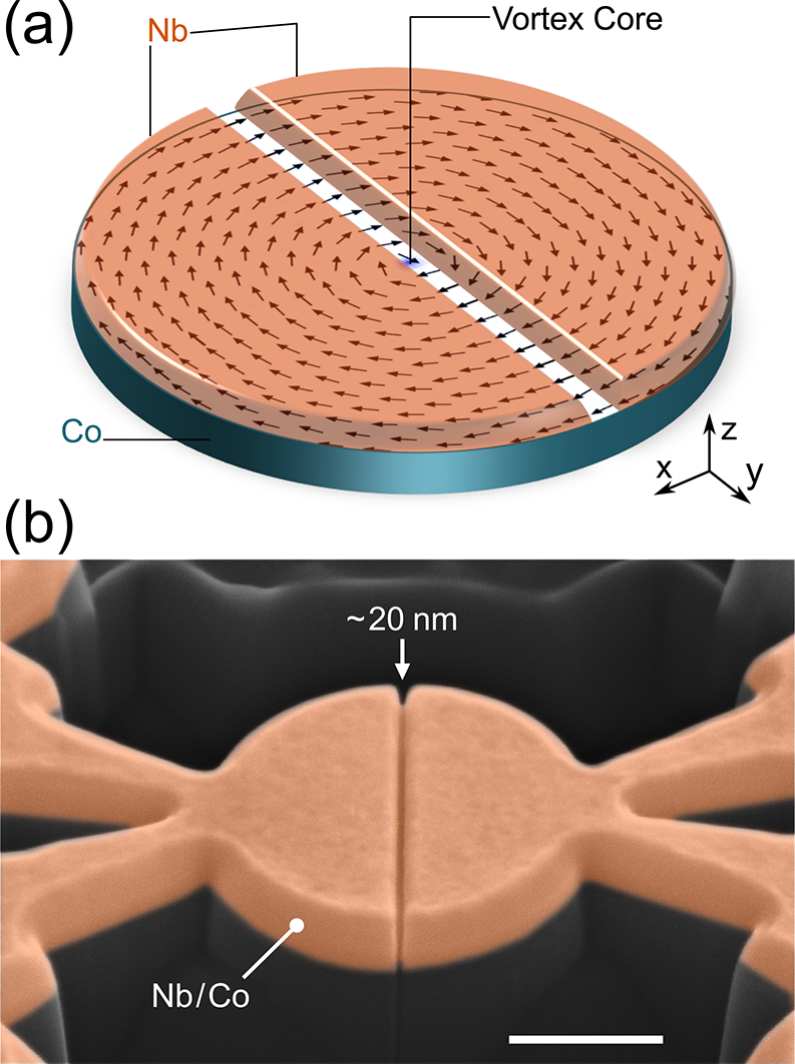
(a) Schematic of the Josephson device. The Nb electrodes are separated by a trench, forming a Co weak link. The pattern on the Co layer corresponds to micromagnetic simulations of a micrometer-size disk (for more information on the micromagnetic simulations, see Supporting Information section S2). The arrows correspond to the in-plane magnetization, while the out-of-plane component is represented by color, which only appears at the vortex core (blue region; less than 5 nm in diameter). (b) False colored scanning electron micrograph of a structured bilayer. The 20 nm gap indicates the Co weak link at the bottom of the trench. The scale bar is equivalent to 400 nm.

To address these issues, we have developed a disk-shaped S–F–S Josephson junction with a highly controllable ferromagnetic vortex spin texture, capable of converting singlet Cooper pairs into LRT currents (see Figure 1). The device consists of a Nb/Co bilayer, where a trench in the Nb layer introduces a (∼ 20 nm) cobalt weak link, which eliminates any singlet or SRT transport. We show that a magnetization gradient of the vortex can act as an effective SOC, which leads to spin accumulation at the rims of the device. This is verified by our transport experiments, which show that the LRT transport is highly localized at the rims of the ferromagnet, resulting in a distinct double-slit supercurrent interference pattern. By modifying the spin texture in a controllable manner, we show that both 0 and π segments can emerge in a single junction. Utilizing the linearized Usadel equation, we examine the microscopic origin of the rim currents in the proximized ferromagnet. Our findings suggest that, in addition to spin texture, superconductor–vacuum boundary conditions play an important role in the singlet to LRT conversion.

## Results and Discussion

### Establishing Long-Range Triplet Transport

**Figure 2:**
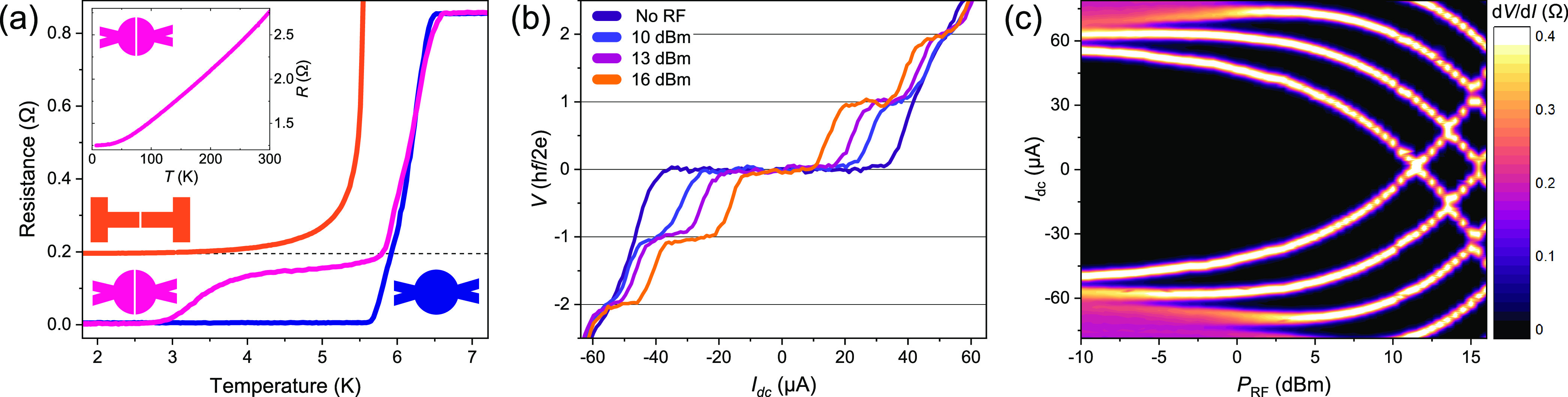
Overview of the basic transport properties of the devices. (a) Resistance as a function of temperature of a proximized disk device (pink) is compared to a Co–Nb disk without a trench (blue) and a bar-shaped device with uniform magnetization (orange). The bar-shaped device is not proximized due to the lack of spin texture (for more information, see Supporting Information section S3). (b) *IV* characteristics at 2 K of a proximized disk device, measured while irradiating the sample with *f* = 1.1 GHz of RF radiation for different powers. The voltage is normalized in units of *hf*/2*e*. (c) Evolution of the Shapiro response as a function of RF power represented as d*V*/d*I* colormap.

Figure 2a shows resistance as a function of temperature for a typical disk junction (see also Supporting Information section S1). A micrometer-wide weak link has a typical resistance of 200 mΩ and becomes fully proximized at low temperatures. We unambiguously establish the Josephson transport in our device by observing their Shapiro response to microwave radiation. This is carried out by measuring the current–voltage (*IV*) characteristics while irradiating the junction with microwaves from a nearby antenna. The *IV* curves show clear Shapiro steps (discrete voltage steps of *hf*/(2*e*), where *h* is the Planck constant, *f* is the frequency, and *e* is the electron charge), which is a result of the phase-locking between the applied microwaves and the Josephson currents (see Figure 2b). We also examined the evolution of the width of the voltage plateaus as a function of microwave power. The results are presented in Figure 2c as a color map of differential resistance.

A direct method to examine the presence of LRT correlations is to verify that once the mechanism for the emergence of LRT pairing is eliminated, the proximity effect will disappear. For instance, in the case of S/F′/F/F′/S multilayer junctions used in previous studies, where the generation of LRT correlations requires a magnetic noncollinearity between the F and F′ layers, the control experiment would show that the critical current (*I*_c_) is heavily suppressed if the F′ layers were either removed or magnetized parallel (or antiparallel) to the F layer.^17−21,60^ The same argument applies here: if the proximity effect is due to LRT correlations produced by the spin texture of the junction, the *I*_c_ must vanish once the magnetization is uniform. This actually happens when we remove the spin texture by applying an in-plane field, typically around 100 mT, which completely suppresses the *I*_c_ (see Supporting Figure S3a). We also verify this through two further experiments, described in Supporting Information section S3. First, we examined the transport in bar-shaped control samples, where shape anisotropy ensures that (even in the absence of in-plane fields) the cobalt layer has a uniform magnetization along the long axis of the bar (see Figure S1). These samples were fabricated via the same procedure as the primary disk-shaped junctions and received the same FIB treatment to structure their weak link. This is evident by the fact that the bar-shaped junctions and the disk devices have a matching barrier resistance (≈ 200 mΩ). Despite multiple attempts, however, the bar-shaped control samples show no sign of long-range proximity. Additionally, we also prepared disk-shaped control junctions where, by applying a lower dose of Ga ions when structuring the weak link, we leave some residual Nb at the bottom of the trench, forming a nonmagnetic channel for singlet transport. Such junctions are completely insensitive to the magnetic state of the cobalt disk and are robust against the in-plane fields used for altering the spin texture; they maintain their *I*_c_ at fields as high as 2 T (see Supporting Information Figure S3b).

### Triplet Currents Confined to the Rims of the Disk

We establish the presence of rim currents using superconducting quantum interferometry (SQI), i.e., measuring the critical current as a function of a magnetic field, applied perpendicular to the transport direction (out-of-plane). Note that the out-of-plane fields used in our SQI experiments are too small to disturb the stable vortex magnetization of the Co disk.

**Figure 3:**
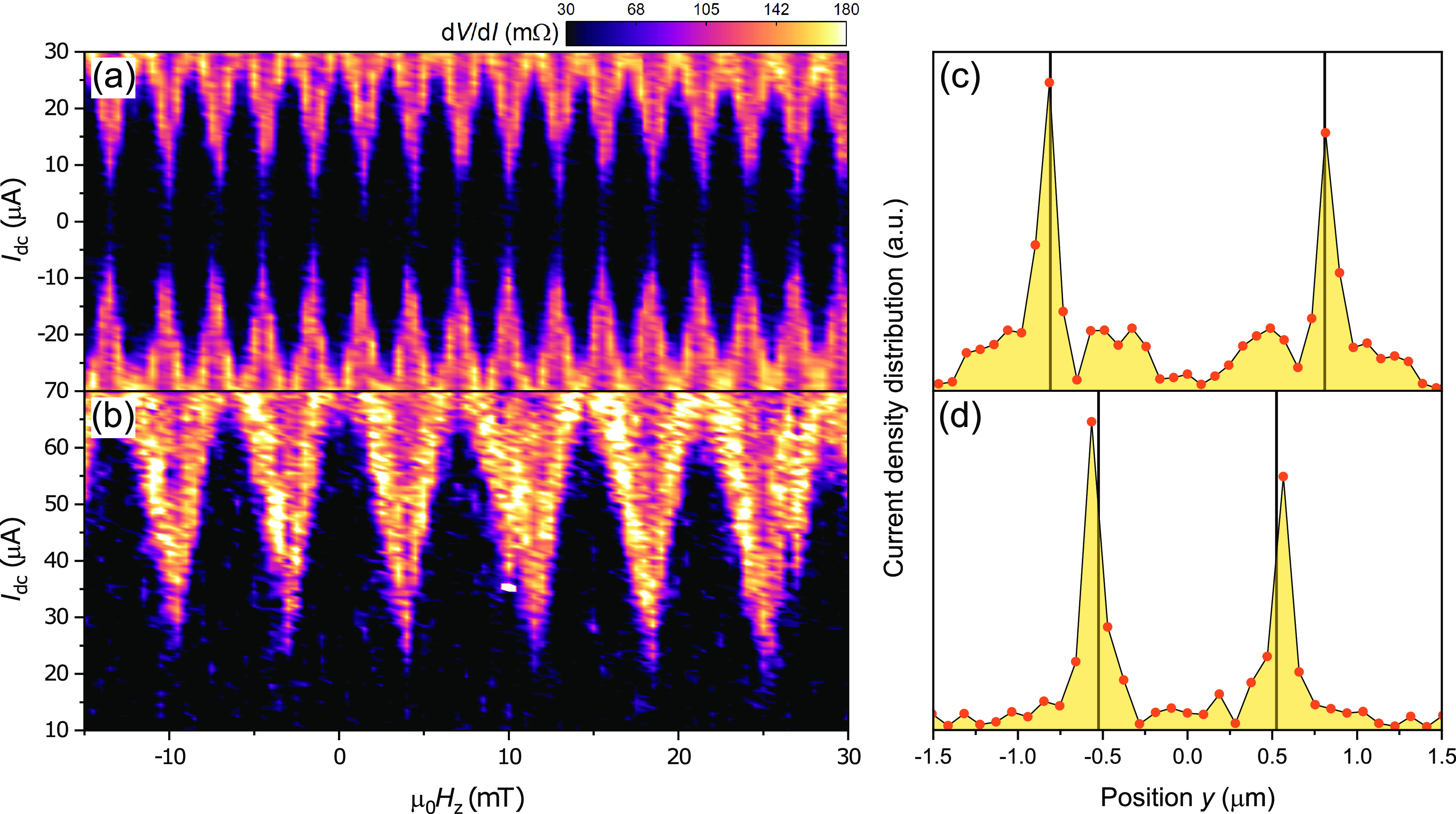
(a, b) Superconducting interference patterns of two junctions with different diameters. The pattern is obtained by measuring the differential resistance as a function of dc current and magnetic field. The disk diameters in (a) and (b) are 1.62 and 1.05 μm, respectively. The period of the oscillations scales inversely with the junction area. In both cases, the junctions show a clear two-channel interference pattern. (c) and (d) depict the critical current density profiles obtained by the Fourier analysis of the patterns in (a) and (b), respectively. The vertical lines indicate the boundaries of the device.

In a conventional junction, the supercurrent is distributed uniformly across the weak link. This results in the well-known Fraunhofer SQI pattern, where the oscillation amplitude has a 1/*B* dependence, and the center lobe is twice as wide as its neighbors. As shown in Figure 3, our devices show a completely different behavior: two-channel interference patterns, characterized by equal-width lobes and slow decay of oscillation amplitude.

All the triplet junctions we measured (over ten devices) show such a two-channel interference pattern. This is illustrated in Figure 3, where we show the SQI patterns for two junctions with different diameters (1.62 and 1.05 μm). Note that the period of the oscillations scales inversely with the area of the junction, which is determined by the radius of the disk. We apply inverse Fourier transform to the SQI patterns to reconstruct the spatial distribution of supercurrent density.^50^ This is a well-established technique, commonly applied to verify the existence of edge currents (see also Supporting Information section S4).^51,52^Figure 3 shows the results of our Fourier analysis for both devices. Regardless of the sample area, we consistently find the supercurrent to be highly localized at the rim of the sample (70 nm or less in width, limited by the resolution of the Fourier analysis). Furthermore, the channels are highly symmetric, as indicated by the sharp cusps of the SQI pattern. Note that the trench is deepest on the sides of the disk (due to a higher milling rate, as can be seen by the small notches on sides of the disk in Figure 1b), making the formation of accidental singlet edge channels even less probable. More importantly, the two-channel behavior is completely absent in all the singlet control samples (see Supporting Information section S3). If the barrier contains residual Nb or is made out of a normal metal (silver), the junction yields a standard single-channel diffraction pattern (Supporting Information Figures S2 and S4b, respectively).

### Altering the Magnetic Texture by an In-Plane Field

**Figure 4:**
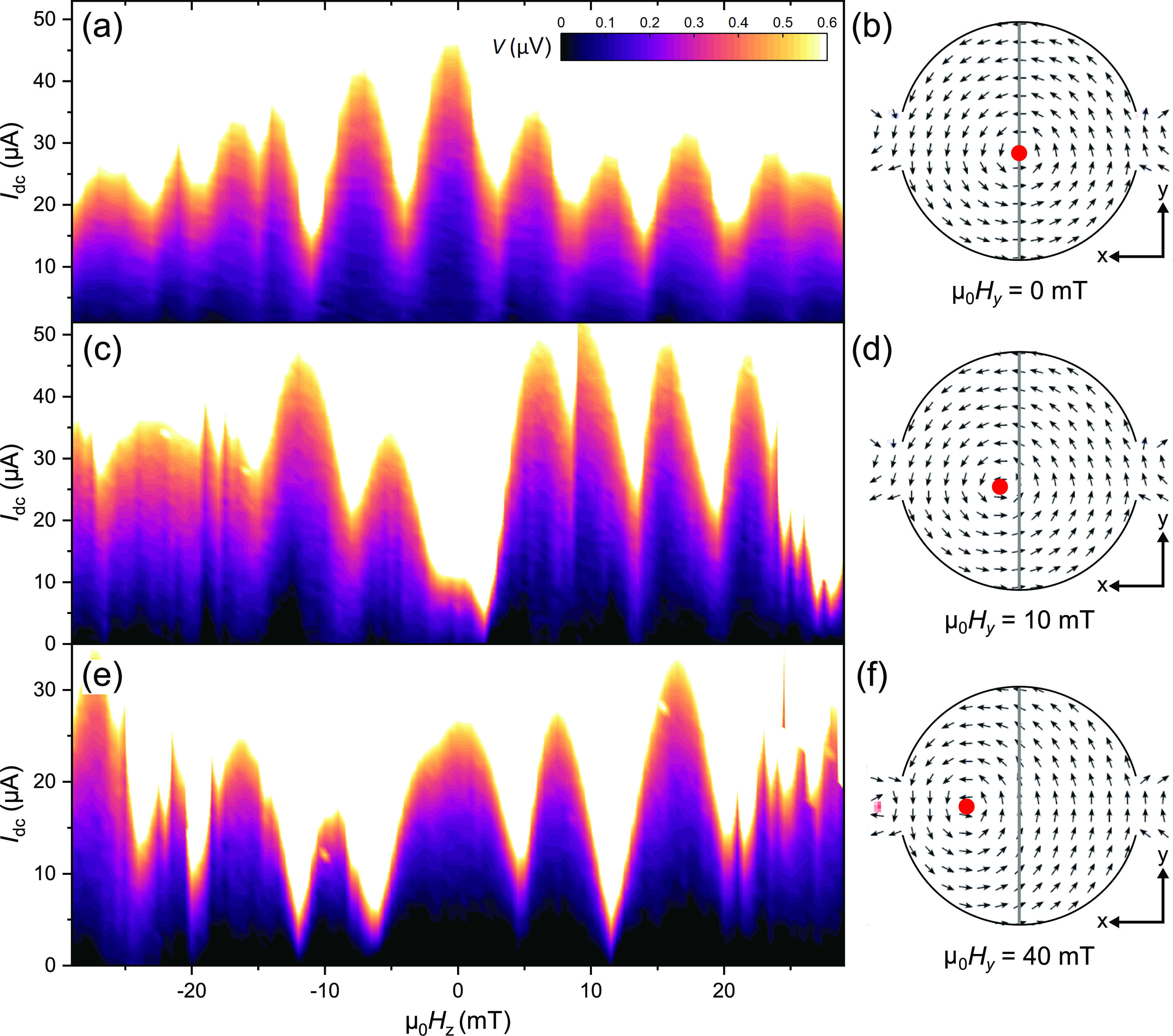
Supercurrent interference patterns (left column) measured at different in-plane fields and the corresponding simulated spin textures (right column). The gray line represents the position of the weak link, and the red dot indicates the location of the vortex core. (a, b) At zero in-plane field, the vortex is at the center of the disk, and a SQUID pattern is observed, i.e., lobes of equal width and slow decay of peak height. (c, d) Applying μ_0_*H*_*y*_ = 10 mT breaks the axial symmetry of the vortex magnetization. This results in the suppression of the middle peak in the interference pattern, characteristic of a 0−π SQUID. (e, f) At μ_0_*H*_*y*_ = 40 mT the vortex core is displaced by over 100 nm. The middle peak of the interference pattern is recovered and its width is doubled with respect to the original pattern. The peak height increases as a function of the out-of-plane field, regardless of sweep direction.

So far we have shown the unconventional distribution of supercurrents through the spin-textured ferromagnetic weak link. We investigate this further by modifying the spin texture using an in-plane magnetic field. As shown in our micromagnetic simulations, in-plane fields can alter the spin texture by effectively moving the vortex core along the axis perpendicular to the field (see Figure 4). For small in-plane fields, the core displacement has an almost linear response and is fully reversible. Using a vector magnet system, we are able to apply a constant in-plane magnetic field while simultaneously acquiring the SQI pattern as described above. Figure 4 shows the SQI patterns measured for different in-plane fields, applied along the trench (*y*-axis), together with the corresponding micromagnetic simulations.

At zero in-plane field (Figure 4a), we observe the aforementioned two-channel (SQUID) interference pattern, with maximum *I*_c_ at zero field. At μ_0_*H*_*y*_ = 10 mT we observe a strong suppression of *I*_c_ for zero out of plane field. Remarkably, however, *I*_c_ is recovered upon increasing the out-of-plane field in either direction. The resulting SQI pattern bears resemblance of a 0−π SQUID: all the lobes are similar in width and *I*_c_ is suppressed around zero. Increasing the *H*_*y*_ to 40 mT, the vortex core has traveled over 100 nm away from the center of the junction (Figure 4e). Interestingly, we find the *I*_c_ to recover for zero out-of-plane field. This reentrant behavior is accompanied by drastic changes to the SQI pattern. The central lobe is now twice as wide, indicating a modified supercurrent distribution, which no longer corresponds to the original two channels. Even more striking is the amplitude of the *I*_c_ oscillations: instead of decaying, the lobes grow taller as we increase the magnitude of the out-of-plane field. This is the universal characteristic of 0−π junctions, i.e., a junction consisting of multiple 0 and π segments connected in parallel.^53−55^ Note that the observed evolution of the interference pattern with the in-plane field cannot be attributed to stray fields or misalignment with the magnet axes since the SQI patterns are independent of magnetic field sweep direction. More importantly, the behavior is completely absent in the control samples with no LRT (Supporting Information section S3), which yield Fraunhofer patterns, regardless of the amplitude or direction of the in-plane field.

### Mapping Spin Texture to Spin–Orbit Coupling

We now continue by describing the mechanism behind the formation of the rim currents using a model that links the vortex spin texture to the SOC. Within the cobalt weak link, the gradient of the local spin texture of the disk junctions is, at the rims of the device, not substantially larger than that of a bar-shaped device. This implies that even though the LRT currents emerge at the rims, they are formed by a process that is sensitive to the global spin texture of the disk.

It was demonstrated that the combination of SOC and exchange field^35,45,47^ or Zeeman field^44,56^ can result in an equilibrium spin current (ESC) which accumulates at the superconducting/vacuum boundaries. We show that a similar process occurs in the presence of spin texture ***m***(*r*), which produces the pure gauge SU(2) field that acts as effective spin–orbit coupling denoted by *iÛ*^†^∇*Û*.^33,34,57^ Here, *Û* (***r***) is the spin-rotation matrix, determined by the transformation to the local spin quantization axis. In the Supporting Information section S5, where we present the full technical details of the Usadel calculations, we show that the vortex spin texture ***m*** = (−sin θ_*v*_ , cos θ_*v*_ , 0) can be transformed into a uniform one, with an effective SOC term *iÛ*^†^∇*Û* = −σ̂_*z*_∇θ_*v*_/2 (here σ̂_*z*_ is the spin Pauli matrix and θ_*v*_ = arctan [(*y*–*y*_*v*_)/(*x*–*x*_*v*_)], where *x*_*v*_ ,*y*_*v*_ are the coordinates of the vortex center). Hence, our system is analogous to one with a uniform magnetization and an intrinsic, spatially inhomogeneous, SOC with the amplitude |∇θ_*v*_| = 1/*r*_*v*_ (with *r*_*v*_ = ) and therefore hosts the aforementioned ESC.

The ESC is carried by the SRT Cooper pairs, which spontaneously appear both at the bottom of the S electrodes and at the top of the F layer. The ESC can be thought of as a spin-imbalance in this SRT condensate. It can be parametrized in terms of the spin vector ***f***, which characterizes the spin component of the triplet condensate. The SRT pairing corresponds to ***f*** = ***m****f*_SRT_, while the LRT one is described by ***f***_LRT_ ⊥ ***m***. The direction of the ESC is determined by the in-plane gradients of the magnetic texture and flows parallel to the S–F interface. In terms of ***f*** it then becomes *J* _*j*_^γ^ ∝ |*f*_SRT_|^2^(***m*** × ∇_*j*_ ***m***)_γ_ (here γ = *x*, *y*, *z* is the index in spin space and *j* = *x*, *y*, *z* is the index in coordinate space). A ferromagnetic vortex texture yields *J* _*j*_^*z*^ ∝ |*f*_SRT_|^2^∇_*i*_ θ_*v*_ = |*f*_SRT_|^2^*m*_*j*_/*r*_*v*_ which is in accordance with the general gauge-invariant expression for the spin current.^58^

### Mechanism for Generating LRT Rim Currents

Having established the equivalency between spin texture and SOC, we now provide a possible mechanism that relates the ESC to the emergence of LRT rim currents (see Supporting Information Figure S7 for a schematic representation). In the absence of spin texture, there is no ESC (Figure S7a). If the spin texture gradient is nonzero, the ESC adiabatically follows the local spin gauge field (***J*** ^*z*^ ∥ ∇θ_*v*_ = (***m*** × ∇***m***)_*z*_; see Figure S7b). When the spin current encounters the bilayer–vacuum boundary (for instance, due to deviations from the ideal circular geometry), the adiabatic approximation breaks down, resulting in an accumulation of spin at the rims of the device. Naturally, the spin accumulation decays over the spin diffusion length, which for cobalt is approximately 60 nm.^59^ Near the interface, the adiabatic ESC can develop a nonzero normal component ***m*** × (*n*_*j*_∇_*j*_)***m*** ≠ 0, where ***n*** is the interface normal. Since the total spin current is zero across the boundary, *n*_*j*_ *J* _*j*_^*z*^ = 0, the adiabatic approximation breaks down and the ESC is compensated by a spin current carried by an LRT condensate, which emerges near the vacuum boundary (Figure S7c). Indeed, considering the local spin basis with ***m*** ∥ ***x*** the vector product of ***f***_SRT_ ∥ ***m*** and ∇_*j*_ ***f***_LRT_ ⊥ ***m*** provides the contribution to the *z*-component of the spin current (mediated by the condensate *J* _*j*_^*z*^ ∼ (***m*** × ∇_*j*_ ***m***)_*z*_) due to the first term of eq 14 in the Supporting Information section S5. This contribution compensates the ESC near the rim. A similar SRT to LRT conversion process has been proposed to occur at the sample boundaries of SNS junctions with intrinsic SOC and a spin active interface^46^ or in one-dimensional systems with a geometric curvature.^47^

**Figure 5:**
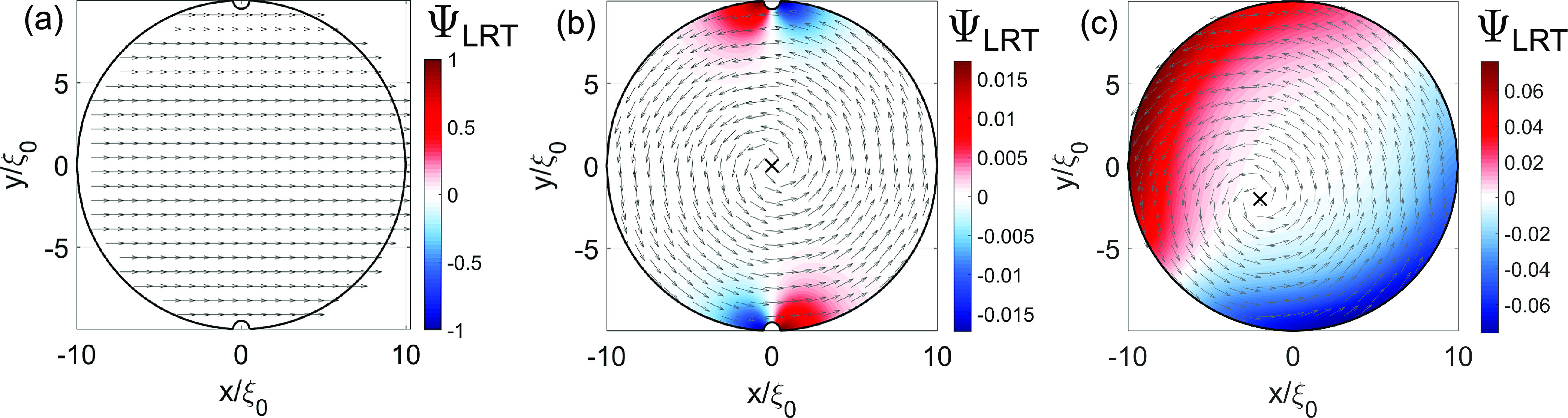
Simulated distribution of Ψ_LRT_ in a S/F bilayer for three different spin configurations, where the amplitude is normalized to (*γ ξ*_0_)*f*_SRT_ (γ is the S–F interface transparency). The direction of the in-plane spin texture is shown by arrows. The simulated geometry includes notches that arise from FIB processing of the actual devices. (a) A homogeneous magnetization, where LRT correlations are completely absent. (b) In the presence of a magnetic vortex, LRT Cooper pairs are generated at the notches, forming two LRT current channels. (c) If the vortex core is shifted from the disk center, an asymmetry arises between the signs of the LRT channels, resulting in 0−π SQUID-like configuration, also in the absence of the notches.

By solving the linearized Usadel equation for a 2D disk-shaped S–F bilayer (without the trench), we simulate the distribution of the LRT amplitude Ψ_LRT_, where ***f***_LRT_ = Ψ_LRT_(*m*_*y*_ , – *m*_*x*_ , 0). The results for three different spin textures are presented in Figure 5. For a uniform magnetization, the LRT correlations are completely absent, regardless of sample geometry (Figure 5a). For a perfectly symmetric vortex pattern, any deviation from the ideal circular geometry at the sample–vacuum boundary (i.e., rim roughness or disorder) results in the emergence of LRT correlations. In our simulation (Figure 5a,b), we use notches on the sides of the disk, also present in our devices, to demonstrate the effect of nonideal boundaries. However, in practice, any deviation from the perfect circular geometry or disorder at boundaries results in a similar outcome. Interestingly, even in the ideal circular geometry with flawless boundaries (i.e., atomically clean and smooth edges), the LRT currents would still appear if the magnetic vortex is not perfectly centered (see Figure 5c). Note that for perfect circular symmetry, with the vortex at the center, *J*_*j*_^*z*^ will always remain parallel to *m*_*j*_ and no LRT is generated.

Our simulations also provide insights into the phase of LRT correlations. When the vortex core is aligned with the trench (at *x* = 0), the LRT currents will result in two π-channels, as indicated by the sign change of Ψ_LRT_. In Figure 5c the vortex is displaced from the center (e.g., due to an in-plane field) and the LRT channels develop opposite signs at the trench. This asymmetry is consistent with the observed 0−π SQUID interference pattern, measured under a constant in-plane field (Figure 4).

### Discussion

While the model presented here can describe the emergence of LRT correlations at the bilayer–vacuum boundaries and the appearance of spontaneous supercurrents in our junctions (i.e., 0−π segments), we should point out that this formalism is restricted to 2D slices of the bilayer. Accounting for the superconductor–vacuum interface formed by the trench is more challenging, as it requires a full three-dimensional model and the knowledge of the exact trench dimensions (e.g., its extent in the Co layer). We discuss this further in Supporting Information section S5.

It should be noted that there is a fundamental difference between the devices presented here and those reported in a previous work, where the disk-shaped junctions consisted of a magnetic multilayer (S–F′–F–F″–S).^15^ In contrast to the Nb/Ni/Cu/Co/Cu/Ni/Nb junctions, where long-range proximity was the result of the magnetic noncollinearity between the Co and Ni layers, here the LRT correlations are generated directly by the spin texture of a single ferromagnet. This is evident by the fact that the multilayer devices were highly sensitive to magnetic conditioning of the Ni (1.5 nm) layer (e.g., the *I*_c_ was irreversibly enhanced when the sample was conditioned), whereas in the case of disks with a single ferromagnet, the transport characteristics are unaltered by magnetic conditioning, since the vortex magnetization is the global ground state of the Co-disk; regardless of the magnetic history, the disk will revert to the vortex magnetization at zero field. This is also confirmed by our micromagnetic simulations. Furthermore, devices with and without the nickel layer exhibit radically different behavior as a function of in-plane fields. However, there are similarities: both devices show a double slit interference pattern, although the current channels are considerably more confined in the case of a single ferromagnetic weak link.

## Conclusions

In summary, we have revealed an unexpected interplay between triplet superconductivity and magnetic texture, which manifests itself as LRT supercurrents localized at the rim of the ferromagnet. We elucidate the origin of the rim currents by mapping the magnetic texture to an effective SOC, which leads to the emergence of equilibrium spin currents, carried by the triplet Cooper pairs present at the S–F interface. We also propose a mechanism for converting the spin currents into equal-spin LRT correlations based on the breakdown of the adiabatic approximation at the sample–vacuum boundaries. Lastly, we show that the nature of LRT transport undergoes drastic changes when the spin texture is modified. As illustrated here, by application of relatively small magnetic fields, the same Josephson junction can be tuned to function as both standard (0–0) and 0−π SQUIDs. The capacity to control supercurrents with the spin texture of a single ferromagnetic layer opens exciting prospects for regulating transport in superconducting devices. Ferromagnetic vortices, in particular, can be manipulated by microwave frequencies in a controllable manner, making them a promising candidate for ultrafast dissipationless electronics.

## Supporting Information Available

Fabrication of the disk devices, micromagnetic simulations, control experiments on disk junctions with a fully nonmagnetic (Ag) barrier, and bar-shaped junctions with a cobalt barrier; description of the Fourier analysis and the full technical details of the Usadel model (PDF)
