# Supplementary material for: Superconducting Triplet Rim Currents in a Spin-Textured Ferromagnetic Disk
Source: Nano Lett. 2022 Mar 3;22(6):2209–16. doi: 10.1021/acs.nanolett.1c04051 (PMC8949790; doi:10.1021/acs.nanolett.1c04051)
Supplement: Supplementary file 1 — nl1c04051_si_001.pdf [file nl1c04051_si_001.pdf]

Supporting Information for: Superconducting triplet rim currents in a spin-textured ferromagnetic disk

Remko Fermin,[1] Dyon van Dinter,[1] Michel Hubert,[1] Bart Woltjes,[1] Mikhail Silaev,[2,3] Jan Aarts,[1] and Kaveh Lahabi[1,†]

[1]*Huygens-Kamerlingh Onnes Laboratory, Leiden University, P.O. Box 9504, 2300 RA Leiden, The Netherlands.*

[2]*Department of Physics and Nanoscience Center, University of Jyväskylä, P.O. Box 35 (YFL), FI-40014 University of Jyväskylä, Finland*

[3]*Computational Physics Laboratory, Physics Unit, Faculty of Engineering and Natural Sciences, Tampere University, P.O. Box 692, Tampere, Finland*

(Dated: February 23, 2022)

† lahabi@physics.leidenuniv.nl

S1. DEVICE FABRICATION

A four-probe contact geometry is patterned on Si substrates, using electron-beam lithography. A cobalt (60 nm) niobium (45 nm) bilayer was deposited in an ultra-high vacuum by Ar-sputtering. The thickness of the Co layer ensures a stable vortex magnetization for the disk diameters discussed in this work (1.05 μm and 1.62 μm). The Co weak link is formed by a line cut through the Nb layer, using an ultra-low beam current of 1.5 pA. The resulting trench separates the Nb electrodes by a $\sim$ 20 nm Co weak link at the center of the disk. A false-colored electron micrograph of a 1.05 μm diameter device is shown in Figure 1b of the main text. To establish a possible link between spin-diffusion length and the width of the rim current channels, several samples, including the one used for Figure 4 of the main text, were deposited with a copper (5 nm) buffer layer, inserted in between the Nb and Co (i.e., after FIB structuring, the layer configuration of the planar junction was Nb/Cu/Co/Cu/Nb). The Cu layer was introduced to enhance the spin-diffusion length at the S-F interface. However, the limited number of lobes in the interference patterns of such devices prevents the Fourier analysis from providing the necessary spatial resolution to quantify the variations in the size of the rim current channel. Qualitatively, no discernible changes could be attributed to the presence of the Cu buffer layer below the Nb electrodes.

S2. MICROMAGNETIC SIMULATIONS

A full description of the micromagnetic simulations of the cobalt layer is reported in a previous work.[1] Within the Object Oriented MicroMagnetic Framework (OOMMF) simulation software, we model the multilayer by dividing it into a three-dimensional mesh of 5 nm cubic cells. The exchange coefficient and saturation magnetization of Co are 3×10^{-11} J/m and 1.4×10^{-6} A/m respectively. The Gilbert damping constant α was set to 0.5 to allow for rapid convergence. In order to represent the polycrystalline nature of the sputtered films, we define the direction of anisotropy by a random vector field.

S3. CONTROL EXPERIMENTS

In this section we establish the vital role of spin texture for generating long range triplet (LRT) correlations. Furthermore, we show that junctions, where transport is dominated by

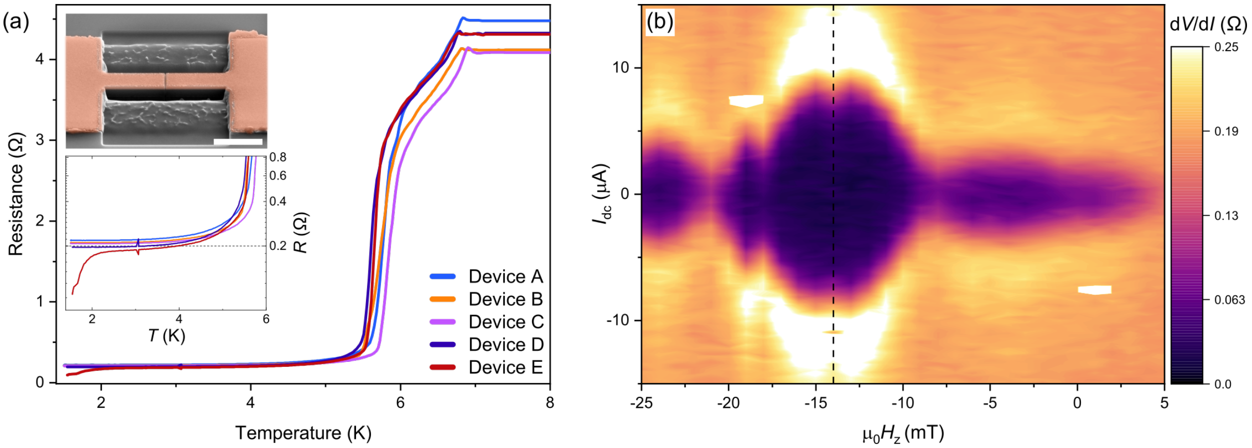

Supporting Figure S1. **a.** Resistance vs temperature obtained on five bar junction devices lacking any spin texture, using a measurement current of 10 μA. The weak link in devices A to D is fabricated following the same method as the triplet disk devices. The low T resistance of these devices equals the weak link resistance (0.2 Ω). As can be seen from the lower inset, only device E shows weak signs of proximity effect, with a heavily suppressed critical current. This device was fabricated using a lower Ga-ion dose, resulting in a Nb-residue in the weak link. The top inset shows a false-colored electron micrograph of a bar junction where the scale bar corresponds to 2 μm. **b.** shows the SQI pattern obtained on device E. The Fraunhofer pattern indicates a uniform current distribution throughout the weak link. The center lobe is shifted to -14 mT due to the magnetic stray fields of the ferromagnet.

singlet correlations, are not affected by the spin texture and that the two-channel behavior is completely absent.

We fabricated control samples with rectangular configuration, where the Co layer is uniformly magnetized along its long axis (*i.e.*, has no spin texture). The bar-shaped devices have equal width (1.05 μm) and layer thicknesses as the triplet disk devices (see top inset of Figure S1a). Due to the 5:1 aspect ratio, no magnetic vortex is stable in the cobalt layer. The same focused ion beam milling procedure was applied to form the weak link. Figure S1a shows the resistance vs temperature of five of such junctions. For devices A to D a similar Ga-ion dose is used for preparing the trench as the triplet disk devices (Nb is completely removed from the weak link). In device E, however, the weak link is fabricated by applying a lower dose, which results in the incomplete removal of the Nb layer in the trench. As shown in Figure S1a, only device E shows signs of short-range proximity effect,

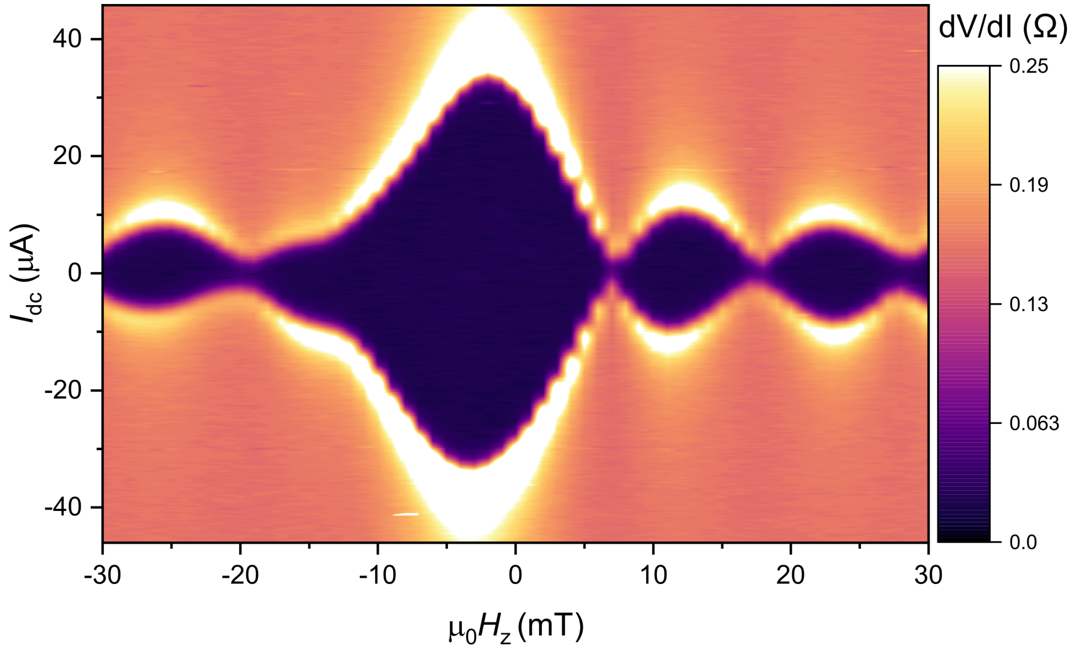

Supporting Figure S2. SQI pattern recorded on a disk-shaped sample containing a shallow trench (*i.e.*, with a Nb-residue in the weak link) and therefore dominated by singlet transport. The central peak is twice as wide as neighboring ones, and the peak height decreases in a $1/B$ fashion. Superconductivity in this sample is found to be unaffected by the removal of the spin texture of the ferromagnet by the application of an in-plane field.

with a heavily suppressed critical current. Figure S1b shows the superconducting quantum interference (SQI) pattern of device E. In contrast to the triplet disk junctions, we observe a distorted Fraunhofer pattern with a rapidly decaying $I_c(B)$, *i.e.*, no two-channel behavior. The pattern is shifted and distorted due to the magnetic dipole fields of the ferromagnet.

We additionally fabricated disk-shaped control samples where the Nb is not completely removed from the weak link to study the interaction of singlet supercurrents with the vortex spin texture. Figure S2 shows an SQI pattern of such a device. Contrary to the triplet devices, we observe a clear Fraunhofer SQI pattern with the central lobe being twice as wide as the subsequent ones and a $1/B$ decay of the peak height. The missing minimum on the negative field side of the pattern may be attributed to the presence of a nearby Abrikosov vortex in one of the superconducting leads.[2] This demonstrates that, despite the ferromagnetic vortex in the Co-layer, the singlet supercurrent is distributed uniformly across the junction. It should also be noted that conventional weak link devices maintain their critical current, even when the spin texture is removed by applying an in-plane field.

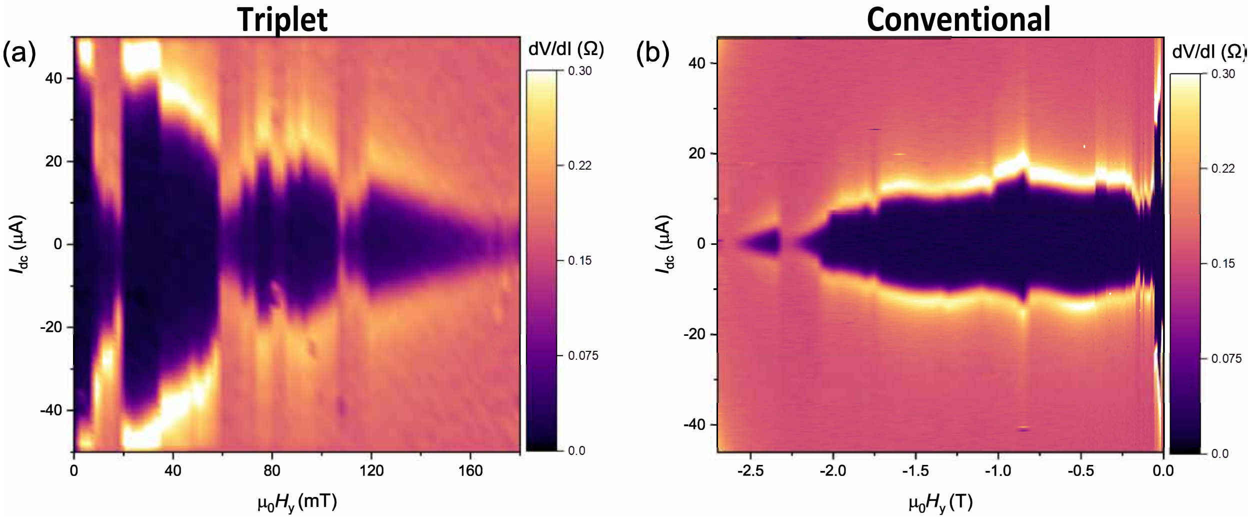

Supporting Figure S3. In-plane $I_c(H_y)$ for a sample with triplet channels and a sample with a conventional weak link. **a.** The triplet sample loses its critical current above 180 mT in-plane field along the trench. At these fields, the spin texture of the disk has become uniform and therefore the generator for LRT correlations is absent. On the sample in **a.** we obtained the data shown in Figure 4 of the main text. **b.** The sample with a shallow trench retains a I_c up to above 2 T, far above the fields required for stabilizing a uniform spin-texture.

In Figure S3 we compare the behavior of a device with triplet channels to that of a device with a shallow trench, i.e., a conventional weak link, under influence of an in-plane field H_y along the trench. The triplet device shows variations in I_c due to 0-π transitions and vice versa, and disappearance of the critical current around 180 mT. The conventional device containing the shallow trench (Figure S2) retains an I_c of around 10 μA up to 2 T.

Finally, in order to verify that the two-channel behavior is not a trivial consequence of the disk configuration, or a byproduct of the FIB milling used in forming the weak link, we examine the Josephson transport of disk-shaped junctions with a non-magnetic barrier. These control samples are structured from a MoGe (55 nm)/Ag (20 nm) bilayer. The Ag weak link is formed through the same FIB treatment as the one applied to the Nb/Co/Nb junctions discussed in the main text. A false colored electron micrograph is shown in Figure S4a and Figure S4b displays an SQI pattern of such device; we find a typical and undistorted Fraunhofer pattern, corresponding to a uniform distribution of supercurrent in the junction. Contrarily to the SF-devices presented in the main text, the shape of the pattern remains unaffected by the application of in-plane fields. To summarize, our control

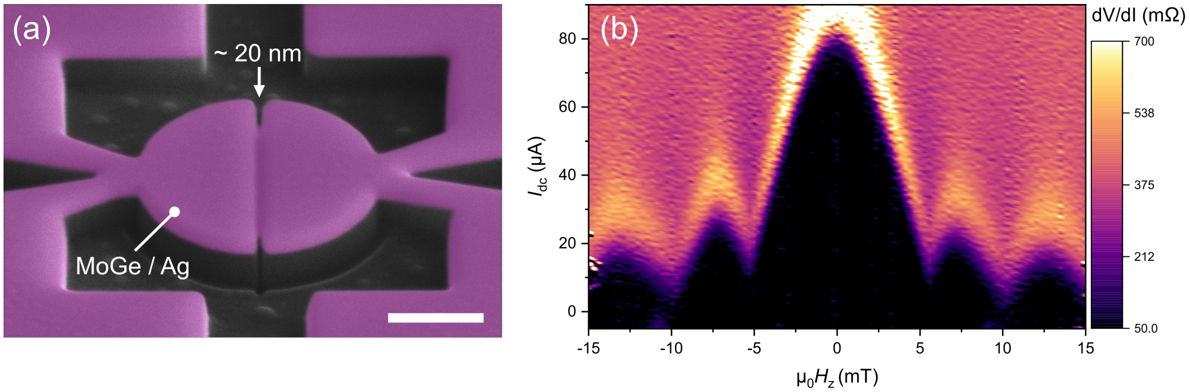

Supporting Figure S4. disk-shaped sample milled out of a Ag/MoGe bilayer and its corresponding SQI pattern. **a.** A false colored electron micrograph of a disk-shaped sample fabricated to have a normal metal weak link. This sample is therefore characterized by singlet transport only. The scale bar corresponds to 500 nm. The SQI pattern displayed in **b.** is a clear Fraunhofer pattern indicating a uniform current distribution, like the case of the shallow trench devices. The pattern is found to be unaffected by the application of in-plane fields.

experiments demonstrate that the rim channels only appear when transport is carried by the LRT correlations. In contrast to singlet or SRT transport, the LRT currents are also highly sensitive to the changes in cobalt spin texture, which are brought about by relatively small in-plane fields.

S4. EXTRACTING THE SUPERCURRENT DENSITY WITH FOURIER ANALYSIS

In their 1971 paper, Dynes and Fulton [3] discuss a relation between the shape of the SQI pattern and the supercurrent distribution in a junction. They realized that, in the case of sinusoidal current phase relation,[4] the current distribution in the junction can be extracted by complex inverse Fourier transform of the field dependence of the critical current. Since the supercurrent can be found from integrating the critical current density along the width of the junction, we can write:

$$I_s(\beta,\phi) = \int_{-W/2}^{W/2} J_s(w)\mathrm{d}w = \int_{-W/2}^{W/2} J_C(w)\sin(\beta w+\phi)\,\mathrm{d}w = \mathrm{Im}\left(e^{i\phi}\int_{-\infty}^{\infty} J_C(w)e^{i\beta w}\,\mathrm{d}w\right) \tag{1}$$

Here w is the position along the junction, W the junction width, $\beta = 2\pi L_{\text{eff}} B/\Phi_0$ is the normalized field (L_{eff} the effective junction length, B the applied field and $\Phi_0 = h/2e$) and ϕ the phase difference at the center of the junction due to a voltage bias to the sample. In the last part, the integration bounds have been extended to infinity since outside the sample there are no supercurrents. The critical current is given by the absolute value of the complex expression:

$$I_c(\beta) = \left| \int_{-\infty}^{\infty} J_C(w) e^{i\beta w} \, \mathrm{d}w \right| = |\Im_c| \tag{2}$$

From this expression a Fourier transform can be recognized where position along the junctions width w and β form conjugate variables. The transform $\Im_c$ is complex and therefore its real and imaginary parts encode for the even and odd components in $I_c(\beta)$ respectively. Since the interference patterns measured in our experiments are relatively symmetric (*i.e.*, an even function of the applied magnetic field), we can assume $\Im_c$ to be dominantly real:

$$I_{c,\text{even}}(\beta) = \int_{-\infty}^{\infty} J_{c,\text{even}}(w) \cos(\beta w) \, \mathrm{d}w \tag{3}$$

Therefore the real part of $\Im_c$ is an oscillating function that flips sign at each zero crossing. The imaginary part is expected to be significantly smaller than the real part, except at the zero-crossing where the even part vanishes. Therefore, the imaginary part of $\Im_c$ ($I_{c,\text{odd}}(\beta)$) can be approximated by the critical current at the minima, flipping sign between each minimum and linearly interpolating between them. The inverse transform yielding $J_C(w)$ from $I_c(\beta)$ is then given by:

$$J_C(w) = \left| \int_{-\infty}^{\infty} (I_{c,\text{even}}(\beta) + iI_{c,\text{odd}}(\beta)) \, e^{i\beta w} \, \mathrm{d}\beta \right| = \left| \int_{-\infty}^{\infty} \Im_c \, e^{i\beta w} \, \mathrm{d}\beta \right| \tag{4}$$

We extract the critical current by a voltage cut-off in each subsequent IV-measurement (*i.e.*, I_c is the current for which we measure a certain voltage over the junction). We observe the minima of the SQI pattern to be non-zero, which might be originating from asymmetries in loop induction or junction capacitance between the two current channels. We vertically translate the extracted I_c values such that the global minimum equals zero current (see figure S5a). This step in the data analysis prevents the overestimation of $I_{c,\text{odd}}(\beta)$, which

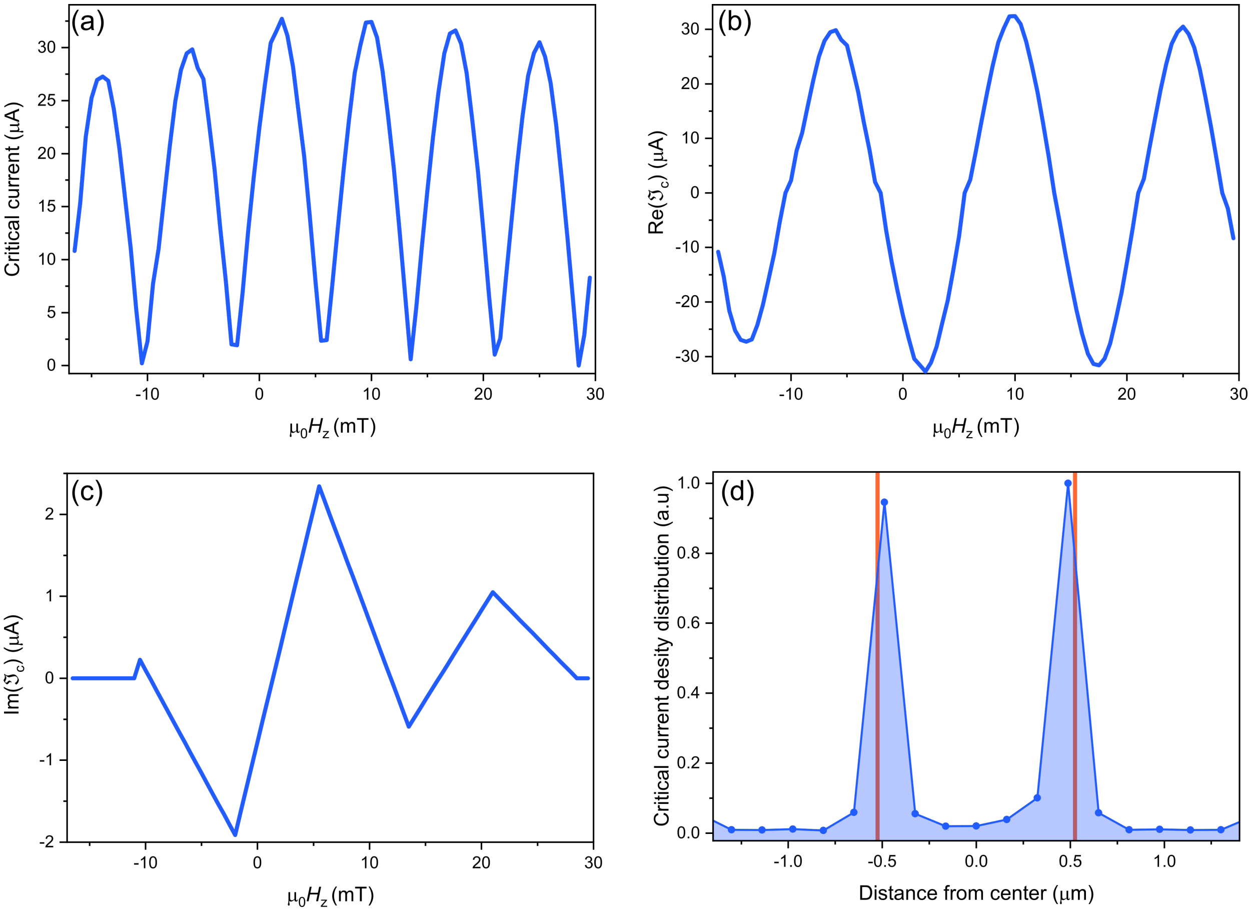

Supporting Figure S5. **a.** Extracted critical current on basis of a voltage cut-off. The global minimum of the interference pattern is shifted to zero current, to not overestimate the imaginary part of the complex critical current. **b.** and **c.** respectively the real and imaginary part of the complex critical current. These are extracted from the interference pattern depicted in a. following the procedure from the main text. **d.** The resulting critical current density distribution calculated using the Fourier transform described in Eq. 4 from the data in b. and c.

would result in an overly anti-symmetric current density distribution. $I_{c,\text{even}}(\beta)$ is found by multiplying the translated I_c by a flipping function that changes the sign of each subsequent lobe of the interference pattern, as can be observed in Figure S5b. We follow the above procedure for finding $I_{c,\text{odd}}(\beta)$, which is depicted in Figure S5c. The corresponding critical current density distribution is found by a numerical Fourier transform carried out in Python using the Numpy package, yielding the distribution in Figure S5d.

The original work of Dynes and Fulton describes an overlap junction between two superconductors that are relatively thick (*i.e.*, the superconductors extend over multiple times λ

on each side of the junction. In this case, both superconductors can effectively shield out the magnetic field, yielding an effective junction length $L = 2\lambda + d$, where d is the barrier thickness. The disk devices, however, are planar junctions with a film thickness that is small in comparison to the London penetration depth ($\lambda(0) = 140$ nm). In this limit, the sine-Gordon equation is modified due to non-local electrodynamics and flux focusing effects, and the period of the SQI pattern is only determined by the geometry of the junction.[5–7] Generally, in this limit $L_{\text{eff}} = 0.56W$ is found. As expected, for our devices, the period of the SQI oscillations scales with the radius of the disk. We find for both radii that:

$$\frac{\Phi_0}{\Delta B(2R)^2} = \frac{\Phi_0}{\Delta B W^2} = 0.26 \tag{5}$$

where ΔB is the field spacing of the peaks in the SQI pattern. The effective length is then determined as:

$$L_{\text{eff}} = \frac{A_{\text{eff}}}{W} = \frac{\Phi_0}{\Delta B W} = 0.26W \tag{6}$$

Resulting in a prefactor of L_{eff} that is significantly lower for our disk junctions. However, if the shielding currents are also restricted in the lateral dimensions, it is shown that the prefactor can decrease.[8] Translated to an equivalent rectangular geometry described in [8], $L_{\text{eff}} = 0.26W$ corresponds to a junction with a total length of $L_{\text{equi}} = 0.7W$. A comparison between such geometry and our disk devices is shown in Figure S6. Evidently, L_{equi} is close to the diameter of the disk, giving a strong indication for the validity of the use of $L_{\text{eff}} = 0.26W$ in the Fourier analysis. The fact that L_{equi} is slightly lower than the diameter can be explained by the circular shape of the sample boundaries, which causes a further restriction to the shielding currents.

It is important to note that the spatial resolution of current density distribution is determined by the number of lobes in the SQI pattern, which is limited by the possible discontinuities in the SQI pattern (typical to junctions with a ferromagnetic weak link). This sets a bound to the precision in determining the width of the current channels. For all measured disk junctions we find the locations of the current channels within 70 nm (binning size of the Fourier transform) from the edges.

Finally, strictly speaking, part of the rectangular junction area that is used in the Fourier analysis falls outside the disk geometry. Using the above-mentioned values of L_{eff}, this

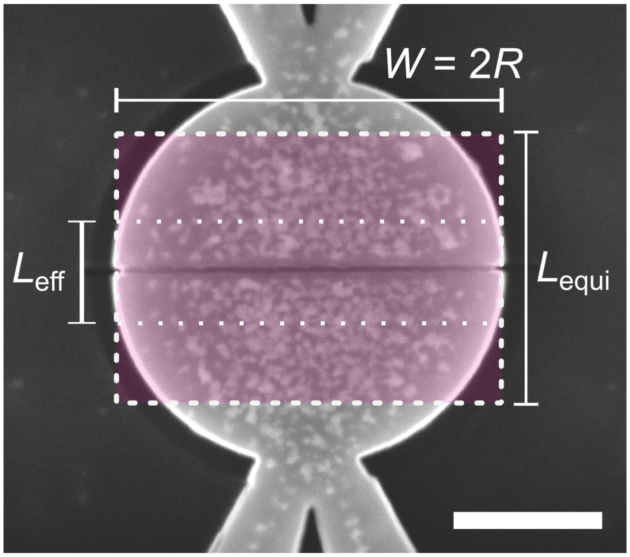

Supporting Figure S6. SEM micrograph of a disk-device ($R = 1.7\ \mu$m), depicted with the length scales relevant to the Fourier analysis. The shaded region corresponds to an equivalent rectangular planar junction, considered in [8], where $L_{\text{equi}} = 0.7W$. $L_{\text{eff}} = 0.26W$ holds both for our disk devices and the equivalent rectangular junction. The scale bar equals 500 nm.

concerns a mere 6% of the total area used in the calculation. Therefore, we can assume the analysis remains valid up to a high degree for our devices.

S5. DETAILS OF THEORETICAL CALCULATIONS

A. Basic equations

To analyze a proximity effect in ferromagnet/superconductor system we use linearised Usadel equation for the anomalous function which takes into account triplet and singlet superconducting correlations. We presented anomalous function in the ferromagnetic region in the form

$$\hat{f} = f_s \hat{\sigma}_0 + i(\boldsymbol{f}\boldsymbol{\sigma}) \tag{7}$$

In this expansion the first term corresponds to the singlet component and the last three terms correspond to the triplet components of the anomalous function. The equations for

components where we include the spin-flip scattering are (for $\omega > 0$):

$$[D\nabla^2 - 2(\omega + 3/\tau_{sf})]f_s + 2(\boldsymbol{h}\boldsymbol{f}) = 0, \tag{8}$$

$$[D\nabla^2 - 2(\omega + 1/\tau_{sf})]\boldsymbol{f} - 2f_s\boldsymbol{h} = 0 \tag{9}$$

with the boundary conditions

$$(\boldsymbol{n}\nabla)f_s|_F = \gamma \hat{f}_s|_S \tag{10}$$

$$(\boldsymbol{n}\nabla)\boldsymbol{f}|_F = \gamma \boldsymbol{f}|_S \tag{11}$$

where γ is the transparency of the F/S interface, D is diffusion coefficient, $\boldsymbol{h}$ is the exchange field proportional to the magnetic moment and ω is the Matsubara frequency. We add the spin relaxation term with rate τ_{sf}^{-1}. The sources in the right-hand side of Eq. 10 and 11 $\hat{f}_s|_S$ and $\boldsymbol{f}|_S$ are the spin-singlet and spin-triplet components of the anomalous function in the superconducting electrode. In the simplest case it contains only the spin-singlet part $f_s|_S = \hat{\Delta}/\sqrt{|\Delta|^2 + \omega^2}$ and $\boldsymbol{f}|_S = 0$. However in general there are also the spin-triplet correlations generated due to the inverse proximity effect [9, 10] or due to the magnetic proximity effect[11–13] through the spin-mixing mechanism.

If the distance between superconducting electrodes is large the current though F is mediated only by the long-range spin-triplet (LRT) components with $\boldsymbol{f}_{LRT} \perp \boldsymbol{m}$, where $\boldsymbol{m}$ is the local magnetization direction.

B. Generation of LRT from spin supercurrent conservation

1. Qualitative picture

Let us consider the overlap geometry when both the S/F interface and the inhomogeneity of the magnetization gradients are in the xy plane. For definitiveness let us assume that magnetization rotates in xy-plane $\boldsymbol{m} = (-\sin\theta_v, \cos\theta_v, 0)$. For example, in the magnetic vortex $\theta_v = \arctan[(y - y_0)/(x - x_0)]$. It is convenient to transform the magnetic texture into the homogeneous one by the spin rotation

$$\hat{f} \to e^{i(\theta_v - \pi/4)\hat{\sigma}_z} \hat{f} e^{-i(\theta_v - \pi/4)\hat{\sigma}_z}; \tag{12}$$

This transformation makes magnetic texture uniform with $m_x = 1$ and $m_z = m_y = 0$. In addition, it leads to the effective spin-orbital coupling (SOC) which shows up in the

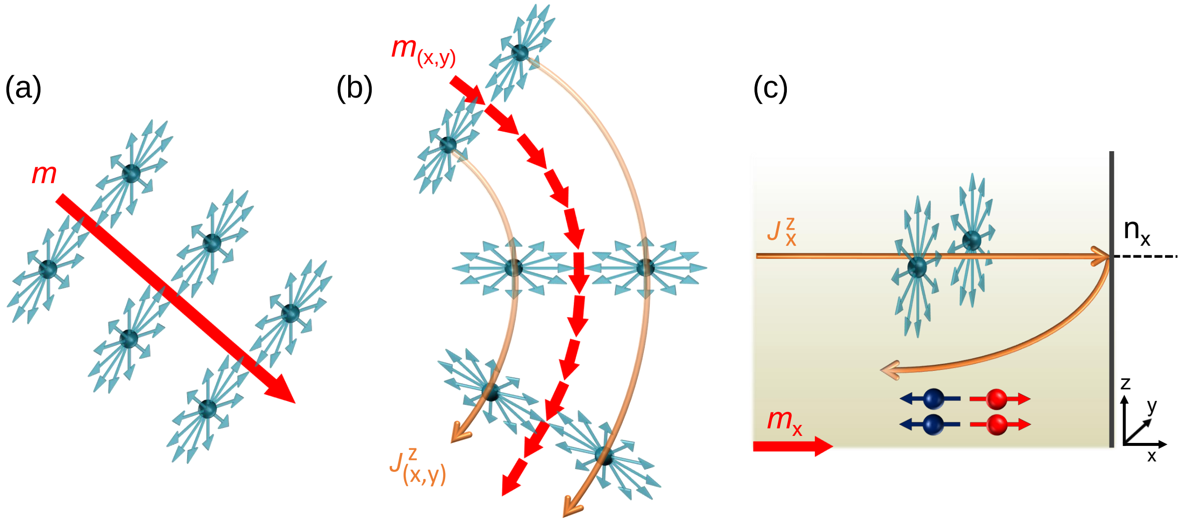

Supporting Figure S7. **a.** SRT Cooper pairs (cyan) in a system with uniform magnetization. The SRT correlations have zero spin projection along the quantization axis, defined by the local magnetization vector $\boldsymbol{m}$ (red arrow). No equilibrium spin current (ESC) or LRT pairs are generated. **b.** In the presence of spin texture, the SRT pairs continuously alter the orientation of their spin plane to remain normal to the local magnetization vector $\boldsymbol{m}_{(x,y)}$, producing an ESC $\boldsymbol{J}^z_{(x,y)}$ (orange arrows), which adiabatically follows the local spin gauge field $\nabla\theta_v = (\boldsymbol{m}\times\nabla\boldsymbol{m})_z$. **c.** When the ESC is incident on a bilayer-vacuum boundary, the adiabatic approximation breaks down. As the ESC cannot propagate outside the bilayer, the total spin current at the boundary must remain zero. To compensate for the adiabatic spin current, a condensate of LRT pairs (in red and blue) with $S_x = \pm 1$ emerges near the vacuum boundary to generate an opposing spin current.

expression for the elongated spatial derivative

$$\nabla_j \hat{f} \to \nabla_j \hat{f} + iZ_j[\hat{\sigma}_z, \hat{f}] = \hat{\boldsymbol{\sigma}}(\nabla_j \boldsymbol{f} - Z_j[\boldsymbol{z}\times\boldsymbol{f}]) \tag{13}$$

with the spin-dependent vector potential $\boldsymbol{Z} = \nabla\theta_v$. The term $\hat{\sigma}_z\boldsymbol{Z}$ plays the role of effective spin-orbital coupling.

The transformation (13) demonstrates the importance of edges in generating the spin-triplet components which are not collinear with magnetization and therefore can penetrate into the ferromagnet. Indeed, far from the magnetic vortex core, the scale of changing

magnetization texture is large, that the emergent vector potential field can be considered as locally-homogeneous $\boldsymbol{Z} = const$ which corresponds to the magnetic texture changing in space with constant gradient. In this case, far from the edges only the correlations with $\boldsymbol{f} \parallel \boldsymbol{h}$ exist in S and in F in the thin layer of the size $\xi_F = \sqrt{D/h}$ near the S/F interface. However even the constant spin-dependent gauge field $\boldsymbol{Z}$ generates the equilibrium spin current [14–16]

$$\boldsymbol{J}_j = \frac{\pi\sigma_n T}{e^2}\sum_\omega (\boldsymbol{f}\times\nabla_j\boldsymbol{f} - Z_j|\boldsymbol{f}|^2\boldsymbol{z}) \tag{14}$$

At the superconductor/vacuum or ferromagnet/vacuum boundaries the spin current should vanish $n_j\boldsymbol{J}_j = 0$. Using (14) this boundary condition can be written as

$$(\boldsymbol{n}\nabla)\boldsymbol{f} = -(\boldsymbol{n}\boldsymbol{Z})[\boldsymbol{z}\times\boldsymbol{f}] \tag{15}$$

It provides the coupling between different spin components of the anomalous function. That is the r.h.s. provides sources of $\boldsymbol{f} \perp \boldsymbol{m}$. Indeed since in the local spin-rotated frame $\boldsymbol{m} \parallel \boldsymbol{x}$ only $\boldsymbol{f} = f_x\boldsymbol{x}$ exist far from the edge. But substituting it to the r.h.s. of (15) we get the sources of the transverse component $\boldsymbol{f}_{LRT} = f_y\boldsymbol{y}$. Note that this boundary condition (15) is valid both in S and F.

The generation of spin-triplet correlations with $\boldsymbol{f}_{LRT} \perp \boldsymbol{m}$ leads to the edge accumulation of the transverse spin component [14–16]. Such correlations can propagate in the ferromagnet at the distances determined by the normal coherence length $\xi_N = \sqrt{D/T}$ or the spin-flip relaxation length $l_s = \sqrt{D\tau_{sf}}$. Therefore we call such correlations as long-range triplet correlations (LRT).

The boundary condition (15) provides the generation of LRT both in the S and F electrodes. The generation in F electrode is described by the linearized Usadel Eqs. (8,9) and boundary conditions (10, 11) with r.h.s. $f_s|_S = F_0 = \Delta/\sqrt{\Delta^2+\omega^2}$ and $\boldsymbol{f}|_S = 0$.

The generation in S electrode is in general determined by the non-linear Usadel equations with inhomogeneous Zeeman field $\boldsymbol{h} \parallel \boldsymbol{m}$ which can be induced either by the inverse proximity effect or the magnetic proximity effect. In the case of inhomogeneous $\boldsymbol{h}$ we again obtain a non-zero LRT source $\boldsymbol{f}|_S \perp \boldsymbol{h}$ in the r.h.s. of the Eq. 11. In both scenarios, the generation of LRT in the presence of a magnetic vortex is described by the qualitatively similar equations derived below; a schematic of the LRT generation is shown in Figure S7.

2. Generation of LRT in S and F electrodes

In general the superconducting correlations both in S and in F are determined by the Usadel equation for the quasiclassical Green function (GF)

$$\nabla(D\hat{g}\nabla\hat{g}) - [\hat{\Delta} + \omega\hat{\tau}_3 + i\boldsymbol{h}\boldsymbol{\sigma}\hat{\tau}_3, \hat{g}] = 0 \tag{16}$$

Here $\hat{\tau}_{1,2,3}$ are the Pauli matrices in Nambu space. The GF has the off-diagonal anomalous part $\hat{\tau}_1\hat{f}$ which describes the superconducting correlations. The field $\boldsymbol{h}$ can be either **(i)** the induced Zeeman field in S or **(i)** the real exchange field inside F. We consider these two cases separately.

(i) First, let us assume that $\boldsymbol{h}$ is the Zeeman field in S, homogeneous over the z coordinate. The local approximation produces the collinear triplet $f_{SRT}\boldsymbol{m}$ which in the simplest case is given by $f_{SRT} = [f_0(\omega+ih) - f_0(\omega-ih)]/2$ where f_0 is the anomalous function in the absence of the Zeeman field. The first-order correction by $\nabla\boldsymbol{m}$ has the form of the transverse spin component $\boldsymbol{f}_{LRT} \perp \boldsymbol{m}$ and appears only because of the boundary condition $\boldsymbol{n}\nabla\hat{g} = 0$ near the edges. The equation is simplified in case that the coplanar magnetization texture $m_z = 0$. Then the transverse spin vector can be found in the form $\boldsymbol{f}_{LRT} = (-m_y, m_x, 0)\Psi_{LRT}$. We assume that the transverse spin-triplet component is small and linearise (16) with respect to Ψ_{LRT}. The amplitude satisfy equations

$$\nabla_\perp^2\Psi_{LRT} - \xi_\omega^{-2}\Psi_{LRT} = 0 \tag{17}$$

$$(\boldsymbol{n}\nabla)\Psi_{LRT} = -\boldsymbol{n}(\boldsymbol{m}\times\nabla\boldsymbol{m})_z\Psi_{SRT} \tag{18}$$

where $\nabla_\perp = (\nabla_x, \nabla_y, 0)$, $\xi_\omega^{-2} = 2(\omega^2 + |\Delta|^2)/D$. The equation is simplified in case of the coplanar magnetization texture $m_z = 0$. Then the transverse spin vector can be found in the form $\boldsymbol{f}_{LRT} = (-m_y, m_x, 0)\Psi_{LRT}$. Solving Eqs.(17,18) we find the distribution of Ψ_{LRT} in the superconductor. Then, using the boundary conditions (11) we obtain the distribution of LRT in the F from the equations

$$\nabla^2\Psi_{LRT} - \lambda_\omega^{-2}\Psi_{LRT} = 0 \tag{19}$$

$$\partial_z\Psi_{LRT}|_F = \gamma\Psi_{LRT}|_S \tag{20}$$

Note that the Eqs.(19) is similar to (17) with the only difference in the decay scale determined by $\lambda_\omega = 1/\sqrt{\xi_\omega^{-2} + \lambda_{sf}^{-2}}$ where $\xi_\omega = \sqrt{D/|\omega|}$ and λ_{sf} is the normal metal spin diffusion length.

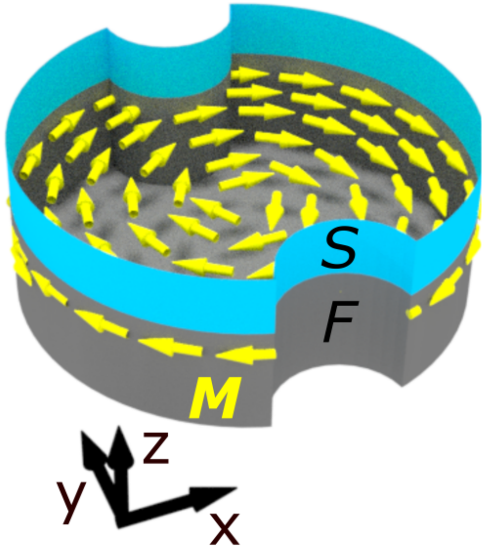

Supporting Figure S8. Sketch of the overlap S/F structure with magnetic vortex pattern in the lower magnetic layer. The notches can be seen on the side.

In case if the F thickness is smaller than the normal metal coherence length and spin diffusion length we can integrate Eqs.(19,20) by z to get the equation with the source

$$\nabla_\perp^2 \Psi_{LRT} - \lambda_\omega^{-2} \Psi_{LRT} = (\gamma/d_F) \Psi_{LRT}|_S \tag{21}$$

Solving this equation we obtain the distributions of LRT show in Figure 5 of the main text.

(ii) The SRT can be generated by the exchange field in F determined by the linearized Usadel Eqs.(8,9). For large exchange field $h \gg T_c$ they have solutions of two types which are the short-range and long-range modes with the scales $\xi_F = \sqrt{D/h}$ and $\xi_\omega = \sqrt{D/|\omega|}$ correspondingly. Hereafter we assume that ξ_F is the smallest length of the problem such that the spatial dependencies of exchange field and geometrical factors are characterized by the scales $\gg \xi_F$.

The spin-triplet correlations can be written as the superposition of short and long-range modes $\boldsymbol{f} = \boldsymbol{f}_{SRT} + \boldsymbol{f}_{LRT}$. The spin vector of short range modes is parallel to the exchange field $\boldsymbol{f}_{SRT} = \boldsymbol{m}\Psi_{SRT}$. Under such conditions we search the short-range solutions of Eqs.(8,9) in the form

$$f_s(\boldsymbol{r}) = -i\gamma\xi_F F_{bcs}(e^{i\pi/4}e^{-\lambda_h z} - c.c.)/2 \tag{22}$$

$$\Psi_{SRT} = -\gamma\xi_F F_{bcs}(e^{i\pi/4}e^{-\lambda_h z} + c.c.)/2 \tag{23}$$

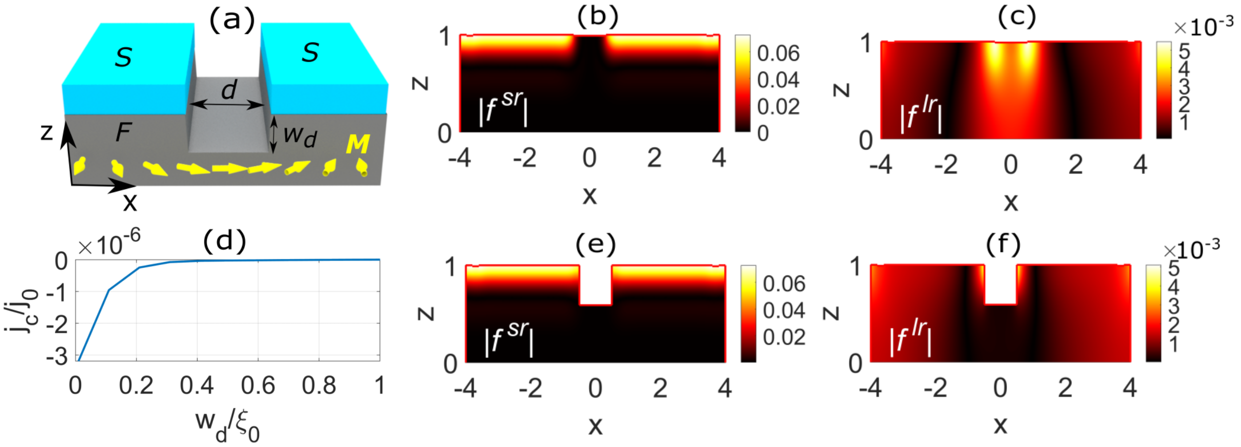

Supporting Figure S9. Spin-triplet superconducting correlations in the model overlap S/F/S junction. (a) Sketch of the setup. The magnetization pattern is $\boldsymbol{M} = M_0\boldsymbol{m}$ where $\boldsymbol{m} = (a, x, 0)/\sqrt{x^2 + a^2}$ and $a = 2\xi_0$. Exchange field is $h = 100T_c$, temperature $T = 0.1T_c$ and spin relaxation rate $\tau_{sf}T_c = 0.25$, the distance between S electrodes is $d = \xi_0$. The phases in $S_{1,2}$ are $\varphi_1 - \varphi_2 = \pi$. The geometric defect in F of the depth w_d is in the trench. (b,c) Distributions SRT. (c,f) Distributions of LRT. (d) Critical current as a function of the depth of the trench w_d.

where the scale is $\lambda_h = e^{i\pi/4}/\xi_F$. In case if the thickness of F is smaller than the coherence and spin diffusion lengths d_F the LRT distribution is determined by the equation similar to Eq. 17

$$\nabla_\perp^2 \Psi_{LRT} - \lambda_\omega^{-2}\Psi_{LRT} = 0 \tag{24}$$

$$(\boldsymbol{n}\nabla)\Psi_{LRT} = -\boldsymbol{n}(\boldsymbol{m} \times \nabla\boldsymbol{m})_z \langle\Psi_{SRT}\rangle_z \tag{25}$$

where the z-averaged SRT is $\langle\Psi_{SRT}\rangle_z = (\gamma/d_F)(T_c/h)F_{bcs}$.

3. Example: LRT generated by the magnetic vortex

The distribution of magnetization in the magnetic vortex far from the vortex core is given by $\boldsymbol{m} = (-sin\theta_v, \cos\theta_v, 0)$ where θ_v is the polar angle with respect to the vortex center. In addition, we take into account the non-circular geometric shape, *e.g.*, shown in Figure S8. We find the LRT distribution solving either the system (17, 18,21) or (24, 25) using finite element numerical package FreeFem[17]. Both systems yield qualitatively similar LRT distributions shown in Figure 5 of the main text.

C. Generation of triplets at the trench

Besides the boundary sources of LRTs, some sources come directly from the Usadel equation in the presence of non-magnetic inhomogeneities such as the order parameter profile.[18] Indeed, the inhomogeneous $|\boldsymbol{f}_{SRT}|^2$ yields the divergence of the second term in the spin current (14) $\nabla_j \boldsymbol{J}_j \propto Z_j \nabla_j |\boldsymbol{f}_{srt}|^2 \boldsymbol{z}$. Such sources have to be compensated by the contribution from the LRT.

To support the above qualitative arguments we consider the generic 2D problem of a superconductor/ferromagnet bilayer shown in Figure S9. We assume that exchange field amplitude is $h = 100T_{c0}$ and the magnetization pattern is $\boldsymbol{M} = M(a, x, 0)/\sqrt{x^2 + a^2}$ with $a = 2\xi_0$. The F-layer has a thickness of ξ_0 and is $8\xi_0$ wide, where $\xi_0 = \sqrt{D/T_c}$.

We consider two model overlap S/F/S junctions shown in Figure S9. The upper row corresponds to smooth edges of S electrodes parametrized by the coordinate-dependent transparency of the S/F interface. The lower row corresponds to the sharp edges of S electrode with $\gamma(x)$ changing abruptly. By solving the linearised Usadel equations (8, 9, 10, 11) with $f_s|_S = \hat{\Delta}/\sqrt{|\Delta|^2 + \omega^2}$ and $\boldsymbol{f}|_S = 0$, using the finite element numerical package FreeFem,[17] we can separate the SRT contribution $\boldsymbol{f}_{SRT} = f_s \boldsymbol{h}/h$ and the LRT one $\boldsymbol{f}_{LRT} = \boldsymbol{f} - \boldsymbol{f}_{SRT}$ for several different configurations. This allows us to study formation of LRT correlations near the edges of F and S layers.

The fourth row showing the total LRT amplitude $|f_{LRT}|$, indicates that they are indeed generated at the boundaries of F layer. Also, LRT correlations are generated near the edges of the overlap S layers which forms the trench. At the same time, this source of LRT generation is very sensitive to the presence of surface defects at the trench. The destructive influence of the defect can be seen in Figure S9 comparing the panels (c) and (f), with a flat surface and the rectangular well between the S electrodes. The suppression of LRT generation results in the strong suppression of the critical current j_c shown in panel (d). The current is normalized to $j_0 = \sigma_n T_c/e$ which is of the order of $10^2/\xi_0$ A/cm. Such current flowing through the cross section of F of the linear size $R \sim 1$ μm yields $j \sim 10^{-8}$ A, which is three orders of magnitude smaller than the experimentally observed value.

[1] K. Lahabi, M. Amundsen, J. A. Ouassou, E. Beukers, M. Pleijster, J. Linder, P. Alkemade, and J. Aarts, Controlling supercurrents and their spatial distribution in ferromagnets, Nat. Commun. **8**, 2056 (2017).

[2] T. Golod, A. Pagliero, and V. M. Krasnov, Two mechanisms of josephson phase shift generation by an abrikosov vortex, Phys. Rev. B **100**, 174511 (2019).

[3] R. C. Dynes and T. A. Fulton, Supercurrent density distribution in josephson junctions, Phys. Rev. B **3**, 3015 (1971).

[4] The disk devices have a sinusoidal current phase relation since they are in the short junction limit.

[5] A. A. Abdumalikov, Jr., G. L. Alfimov, and A. S. Malishevskii, Nonlocal electrodynamics of josephson vortices in superconducting circuits, Superconductor Science and Technology **22**, 023001 (2009).

[6] M. Moshe, V. G. Kogan, and R. G. Mints, Edge-type josephson junctions in narrow thin-film strips, Phys. Rev. B **78**, 020510(R) (2008).

[7] A. A. Boris, A. Rydh, T. Golod, H. Motzkau, A. M. Klushin, and V. M. Krasnov, Evidence for nonlocal electrodynamics in planar josephson junctions, Phys. Rev. Lett. **111**, 117002 (2013).

[8] J. R. Clem, Josephson junctions in thin and narrow rectangular superconducting strips, Phys. Rev. B **81**, 144515 (2010).

[9] F. Bergeret, A. F. Volkov, and K. Efetov, Induced ferromagnetism due to superconductivity in superconductor-ferromagnet structures, Phys. Rev. B **69**, 174504 (2004).

[10] F. Bergeret, A. L. Yeyati, and A. Martin-Rodero, Inverse proximity effect in superconductor-ferromagnet structures: From the ballistic to the diffusive limit, Phys. Rev. B **72**, 064524 (2005).

[11] T. Tokuyasu, J. A. Sauls, and D. Rainer, Proximity effect of a ferromagnetic insulator in contact with a superconductor, Phys. Rev. B **38**, 8823 (1988).

[12] A. Cottet, D. Huertas-Hernando, W. Belzig, and Y. V. Nazarov, Spin-dependent boundary conditions for isotropic superconducting green's functions, Phys. Rev. B **80**, 184511 (2009).

[13] M. Eschrig, A. Cottet, W. Belzig, and J. Linder, General boundary conditions for quasiclassical theory of superconductivity in the diffusive limit: application to strongly spin-polarized

systems, New J. Phys. **17**, 083037 (2015).

[14] M. Alidoust and K. Halterman, Spontaneous edge accumulation of spin currents in finite-size two-dimensional diffusive spin–orbit coupled SFS heterostructures, New J. Phys. **17**, 033001 (2015).

[15] M. Alidoust and K. Halterman, Long-range spin-triplet correlations and edge spin currents in diffusive spin–orbit coupled SNS hybrids with a single spin-active interface, J. Condens. Matter Phys. **27**, 235301 (2015).

[16] I. V. Tokatly, B. Bujnowski, and F. S. Bergeret, Universal correspondence between edge spin accumulation and equilibrium spin currents in nanowires with spin-orbit coupling, Phys. Rev. B **100**, 214422 (2019).

[17] F. Hecht, New development in freefem++, J. Numer. Math. **20**, 251 (2012).

[18] M. S. Kalenkov, A. D. Zaikin, and V. T. Petrashov, Triplet superconductivity in a ferromagnetic vortex, Phys. Rev. Lett. **107**, 087003 (2011).
